# A preliminary evaluation of influence of body mass index on in vitro fertilization outcome in non-obese endometriosis patients

**DOI:** 10.1186/s12905-017-0457-0

**Published:** 2017-11-16

**Authors:** Eliana Garalejic, Biljana Arsic, Jovana Radakovic, Dragana Bojovic Jovic, Dragana Lekic, Biljana Macanovic, Ivan Soldatovic, Milan Perovic

**Affiliations:** 1 IVF Department, Clinic for Gynecology and Obstetrics “Narodni front”, Kraljice Natalije 62, Belgrade, 11000 Serbia; 2 School of Medicine, University of Belgrade, Dr Subotica 8, Belgrade, 11000 Serbia; 3 Institute of Medical Statistics and Informatics, Dr Subotica 15, Belgrade, 11000 Serbia; 4 Faculty of health, legal and business studies, Singidunum University, Zeleznicka 5, Valjevo, 14000 Serbia

**Keywords:** Endometriosis, Body mass index, In vitro fertilization, Pregnancy rate

## Abstract

**Background/aims:**

Obese and overweight women experience a lower probability for pregnancy after IVF. However, despite the increasing prevalence of obesity, the large majority of infertile women are non-obese. One of the most common indications for IVF is endometriosis. Thought-provoking inverse correlation has been established between BMI and endometriosis. Lower BMI is a risk factor for development of endometriosis and a predictive factor for severe endometriosis. Since severe endometriosis carries lower reproductive chances, even after IVF, we preliminary tested a hypothesis that higher BMI among non-obese endometriosis patients improves IVF outcomes.

**Methods:**

Preliminary retrospective observational cross-sectional study was performed in women with endometriosis as a sole infertility cause who underwent IVF. During analyzed period we performed 2782 IVF procedures. In order to achieve highly homogenous study sample and to eliminate almost all confound factors that could lead to bias, we implemented strict study criteria. The number of eligible subjects was 156 and they were divided into underweight, normal weight and overweight groups. Primary outcomes were number of retrieved oocytes, good quality oocytes, embryos, and the rates of biochemical, clinical and ongoing pregnancies. For group comparisons, we used parametric test, analysis of variance, and non-parametric tests (Kruskal-Wallis test, Chi-square test). Logistic regression and General linear model was used to assess correlation between BMI and dependent variables (outcome and stimulation duration) when adjusted for age.

**Results:**

Endometriosis as a single infertility factor among IVF couples had prevalence of 5.61%. Underweight women accounted for 10.26%, normal weight 71.15% and overweight 18.59% of study population. Significant differences were not found in number of retrieved oocytes (*p* = 0.880), good quality oocytes (*p* = 0.476), obtained embryos (*p* = 0.706), and biochemical (*p* = 0.298), clinical (*p* = 0.770) and ongoing (*p* = 0.822) pregnancy rates between study groups.

**Conclusion:**

Although preliminary results do not support our hypothesis, increase in BMI did not adversely affect the outcome of IVF in non-obese endometriosis patients, which is in contrast to literature data as regards general population of infertile women undergoing IVF. Prospective studies with large number of patients with endometriosis or prospective case-control studies should address these issues and provide more comprehensive counseling of infertile endometriosis patients regarding achievement of optimal BMI prior to IVF with the intention of achievement higher pregnancy rates.

## Background

Following the publication of numerous studies which demonstrated sub-optimal reproductive ability of obese women, the consequences of the obesity on in vitro fertilization (IVF) have been in the focus of the contemporary infertility research. Overweight and obese women experience a lower probability for pregnancy after IVF [1]. Body mass index (BMI) is inversely related to intrafollicular human chorionic gonadotropin (hCG) concentrations, embryo quality and IVF outcome [2]. Despite the increasing prevalence of obesity, obese women make up a lesser proportion of women who obtain fertility-related services and the large majority of infertile women are still in the non-obese BMI range [3]. Against this background, it would be important to evaluate the influence of BMI on IVF outcome among the non-obese women.

Therewithal, thought-provoking relationship has been established between BMI and the endometriosis, the latter being one of the most common causes of infertility. Endometriosis demonstrated an inverse correlation with BMI as infertile obese women were at lower risk for development of endometriosis [4]. Furthermore, it has been shown that lower BMI could be considered as a predictive factor not only for any type of endometriosis but also for severe ones [5]. The fact that moderate and severe forms of the disease carry lower reproductive chances lead us to a hypothesis that higher BMI among endometriosis patients increases the probability of conception during IVF treatment.

Taking into account that, to the best of our knowledge, the impact of BMI on the outcome of IVF in non-obese women with endometriosis has not yet been evaluated, the aim of our study was to perform preliminary assessment of the influence of BMI on IVF outcome in these patients and to provide up-to date data for future trials in this field. Considering that vast majority of endometriosis patients are not obese, characterizing association of a range of BMIs with IVF outcome could enlighten the management of endometriosis patients.

## Methods

We investigated the influence of BMI on outcome of first, fresh, autologous IVF cycles in non-obese patients (BMI < 30) with previously diagnosed endometriosis in an observational cross-sectional study within IVF Department of The University Clinic for Gynecology and Obstetrics “Narodni front”, Belgrade, from 1st January 2007 to 31st May 2016. This study was approved by the Institutional review board (decision No.24/10–3).

Medical records in our department before 2011 were kept in paper format. After 2011, the records were kept both in paper and electronic medical records (Meditex software, Fertility database system for therapy documentation and quality assurance for reproductive medicine, CRITEX GmbH, Regensburg, Germany). All data have been entered in electronic records exclusively by gynecologists and embryologists of our IVF department, who are also the members of the study team (BA, DL, DBJ, BM, MP) and the data before 2011 have been entered retrospectively. The study data came from electronic medical records and lifestyle and exposure factor questionnaires completed during the admission to our department. The study data were extracted by MP, BA, DBJ, DL, BM and JR and validated by the Head of IVF Department (EG).

We analyzed the data of laparoscopically diagnosed patients with any of all four grades of the endometriosis according to the Revised Classification of the American Society of Reproductive Medicine (ASRM) [6]. The endometriosis staging at our clinic is performed at the time of laparoscopy and reassessed immediately after operation by senior doctors who are calculating a score from reading the operative notes. We investigated only infertile women who underwent laparoscopy. Therefore the basic criteria for performing laparoscopy in our study participants were infertility or infertility with the assumption of the existence of endometriosis on the basis of previous clinical and ultrasound findings.

A standardized infertility evaluation was performed on all study participants. In order to disable the effects of male infertility on IVF outcome, we investigated only women whose partners had normal semen analysis [7]. With the aim of avoidance of influences of other female infertility factors on the outcome of IVF, we analyzed the data of women with endometriosis as the only cause of infertility. Furthermore, having in mind that the surgical procedures performed on the ovaries and fallopian tubes compromise the vascularity of the ovary and thereby may affect ovarian reserve and reproductive chances, women with this kind of operations unrelated to the treatment of endometriosis, were also excluded. Additionally, women with diseases which could influence the IVF outcome (i.e. autoimmune disorders, diabetes mellitus, trombophylias, thyroid gland diseases), were excluded too.

Study participants were divided into underweight (BMI < 18.5), normal weight (BMI 18.5–24.9), and overweight (BMI 25–29.9) groups. Calculation of BMI was performed from anthropometric measurements of patients obtained in our department. Patients were measured on a digital scale that shows body weight in kilograms by the reliability of ±100 g. Measurement of body height was done in a standing position, without shoes, with shoulders in a relaxed position and was measured in cm and measured to the nearest 0.5 cm tick marks.

Several characteristics of study participants were analyzed to eliminate possible confounding factors influencing the outcome of IVF. Evaluated demographic characteristics were age, race, marital status and educational level. Analyzed clinical features of study patients encompassed markers of ovarian reserve, such as Anti-Müllerian hormone (AMH), basal serum concentrations of estradiol (E_2_) and follicle-stimulating hormone (FSH), antral follicle count (AFC), duration of infertility prior to IVF procedure and type of infertility (primary vs. secondary), type of stimulation protocols and endometriosis grade according to the ASRM. Environmental exposures and life style factors that may affect IVF outcome were assessed as well. Evaluated life style factors were caffeine intake, alcohol and marijuana consumption and exercise. Apprised environmental and occupational exposures were video display terminals, frequent changes in time zones in the workplace, exposure to noise, psychological stress in the workplace, constant exposure to toluene, xylene, formalin, chloroform, ethylene glycol ethers, antineoplastic agents (i.e. dental surgeons, pharmacy and nursing staff, workers in biomedical research laboratories).

We used Gonadotropin-releasing hormone (GnRH) agonist (GnRH-a) and GnRH antagonist (GnRH-an) protocols for controlled ovarian stimulation (COS) during the study period. Protocol type was chosen for each patient individually, based on the benefits and shortcomings of each treatment option, and more significantly on the patients’ response and characteristics. Usually, GnRH-an protocol was used for patients with high risk for developing ovarian hyperstimulation syndrome, patients with a higher number of previously unfavorable cycles and patients with advanced age. The presence of at least three follicles with diameter > 17 mm measured during transvaginal ultrasound scan was the criterion for triggering. In case of poor ovarian response, final oocyte maturation was induced if there had been only one follicle with diameter > 17 mm. Ultrasound guided aspirations were performed 35 h after triggering. Two, three or 5 days after oocyte retrieval, embryotransfers (ET) of only top and good quality (GQ) embryos were performed. Micronized Progesterone at daily dose of 600 mg and 250 mg of Hydroxyprogesterone caproate every fifth day, were used for the luteal support.

The analyzed parameters were: duration of COS; the dose of applied gonadotropins; total number of oocytes and number of GQ oocytes obtained by aspiration of follicles; total number of embryos, number of GQ embryos and number of transferred embryos; the rates of biochemical, clinical and ongoing pregnancies. Under the oocytes of good quality we have assumed metaphasis II oocytes and fertilized oocytes after IVF. Embryos were stratified to top, good, poor and bad according to the Instanbul consensus workshop on embryo assessment [8]. Serum β-hCG >50 mIU/ml measured on the 16th day after oocyte retrieval were considered as biochemical pregnancy. Clinical pregnancy was defined as pregnancy with gestational sac or fetal pole visualized on an early ultrasound examination at the 7th week of gestation. The ongoing pregnancy was defined as vital pregnancy with normal ultrasound findings in the 12th week of gestation. All pregnancy rates were calculated per ET.

Data are presented as counts (percents) or mean+/−standard deviations, depending on data type. For group comparisons, we used parametric test, analysis of variance (ANOVA), and non-parametric tests (Kruskal-Wallis test, Chi-square test). Logistic regression and General linear model was used to assess correlation between BMI and dependent variables (outcome and stimulation duration) when adjusted for age. All data analysis was performed using the statistical software SPSS (IBM corp.). All *p* values less than 0.05 were considered significant.

## Results

During the study period, we performed IVF procedure in 2782 women. After implementation of study criteria, the total number of eligible subjects was 156. Among them 16 were underweight (10.26%), 111 had normal weight (71.15%) and 29 were overweight (18.59%). Characteristics of study patients are presented in Table 1. Highly significant differences were found in age, exercise, educational level and place of residence. None of the study subjects reported alcohol and marijuana consumption.

**Table 1:** Characteristics of the study population^a^

	Body mass index groups			*p* value
	Underweight	Normal weight	Overweight	
BMI (kg/m^2^)	17.86 ± 0.51	22.08 ± 1.61	26.77 ± 1.49	NA
Age (years)	31.25 ± 2.59	34.25 ± 3.42	35.17 ± 3.31	0.001^†^
Marital status	12 (75%)	90 (81.1%)	21 (72.4%)	0.551^‡^
Educational level				
Primary school	1 (6.25%)	7 (6.3%)	6 (20.68%)	0.001^‡^
Secondary school	8 (50%)	55 (49.54%)	21 (72.41%)	
University level	7 (43.75%)	48 (43.24%)	2 (6.89%)	
Place of residence				
Rural population	1 (6.2%)	14 (12.6%)	10 (34.5%)	<0.010^‡^
Urban population	15 (93.8%)	97 (87.4%)	19 (65.5%)	
Cigarette smoking	8 (50%)	26 (23.42%)	9 (31.03%)	0.076^‡^
Caffeine intake	10 (62.5%)	70 (63.06%)	15 (51.72%)	0.532^‡^
Exercise	2 (12.5%)	23 (20.72%)	0 (0%)	0.019^‡^
Occupational exposure	1 (6.25%)	5 (4.5%)	0 (0%)	0.434^‡^
Environmental exposure	1 (6.25%)	3 (2.7%)	0 (0%)	0.613^‡^

^a^Data are presented as mean ± SD or counts (%). ^†^ANOVA. ^‡^Chi-square test. *NA* non applicable

Analyzed clinical characteristics of participants which may influence the IVF outcome are presented in Table 2. Regarding AMH and AFC, data were known for 109 patients, since the assessment of ovarian reserve with AMH and AFC was not obligatory practice at our clinic until year 2010. Significant differences among study groups were found in previous surgical treatment of endometriosis, as expected. As BMI increased, the percentage of previously surgically treated patients decreased.

**Table 2:** Clinical characteristics of study population^a^

	Body mass index groups			*p* value
	Underweight	Normal weight	Overweight	
Basal E_2_ (pmol/ml)	191.58 ± 104.97	194.79 ± 180.48	220.56 ± 177.41	0.779^†^
Basal FSH (mIU/ml)	7.22 ± 2.95	7.65 ± 2.86	7.22 ± 3.84	0.259^†^
AMH (ng/ml)	1.46 ± 1.21	1.84 ± 1.61	2.21 ± 1.76	0.447^†^
AFC	9.8 ± 3.51	11.6 ± 6.03	10.76 ± 5.32	0.742^†^
Infertility duration	6.5 ± 3.35	7.03 ± 3.32	8.18 ± 4.2	0.272^†^
Endometriosis				
Grade I	2 (12.5%)	33 (29.73%)	16 (55.17%)	<0.001^§^
Grade II	2 (12.5%)	24 (21.62%)	9 (31.03%)	
Grade III	7 (43.75%)	41 (36.94%)	4 (13.80%)	
Grade IV	5 (31.25%)	13 (11.71%)	0 (0%)	
Infertility type				
Primary	14 (87.5%)	88 (79.27%)	21 (72.4%)	0.484^‡^
Secondary	2 (12.5%)	23 (20.72%)	8 (27.58%)	
Surgical treatment for endometriosis				
None	1 (6.25%)	28 (25.22%)	13 (44.82%)	0.048^‡^
Electrocoagulation	4 (25%)	12 (10.81%)	7 (24.13%)	
Cystectomy	4 (25%)	41 (36.93%)	5 (17.24%)	
Bilateral cystectomy	3 (18.75%)	9 (8.1%)	1 (3.44%)	
Cystotomy	2 (12.5%)	12 (10.81%)	1 (3.44%)	
Bilateral cystotomy	2 (12.5%)	9 (8.1%)	2 (6.89%)	
Endometrioma				
None	9 (56.25%)	72 (64.87%)	23 (79.31%)	0.083^‡^
Unilateral	4 (25%)	33 (29.73%)	6 (20.69%)	
Bilateral	3 (18.75%)	6 (5.40%)	0 (0%)	

^a^Data are presented as mean ± SD or counts (%). ^†^Kruskal-Wallis test. ^‡^Chi-square test. ^§^Chi-square test for trend

**Fig. 1:**
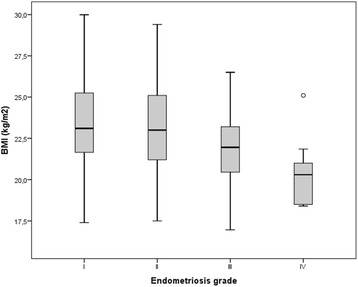
Correlation of BMI and Stage of Endometriosis

The revealed differences among study groups in frequencies of different grades of endometriosis were significant (*p* < 0,001), as shown in Table 1. Highly significant negative correlation (p < 0,001) was found between BMI and the grade of the endometriosis, with mean BMI being the highest in the minimal form of the disease (ASRM grade I) and the lowest in the severe form (ASRM grade IV), which is shown in Fig. 1.

We used GnRH-a protocol in 68 patients, while GnRH-an protocol was applied in 88 patients. Cycle cancellation due to insufficient follicular development was present in four patients (2.56%), which included three participants in normal weight group and one in overweight group. Although they reached the ovum pick-up stage, two patients (1.28%) were without retrieved oocytes after the procedure. Consequently, primary study outcomes were analyzed in 150 women. The other characteristics of IVF treatment and outcome according to BMI are presented in Table 3.

**Table 3:** Characteristics of IVF treatment and outcome according to BMI^a^

	Body mass index groups			*p* value
	Underweight	Normal weight	Overweight	
No of started cycles	16 (10.26%)	111 (71.15%)	29 (18.59%)	NA
Stimulation duration (in days)	10.31 ± 1.08	9.83 ± 1.48	10.83 ± 2.05	0.009^†^
GnRH-a	6 (37.5%)	50 (45.04%)	12 (41.37%)	0.821^‡^
GnRH-an	10 (62.5%)	61 (54.96%)	17 (58.63%)	
E_2_ on triggering day (pmol/ml)	6.158 ± 2.836	5.673 ± 2.769	5.244 ± 2.876	0.599^†^
Total gonadotropin dose (IU)	3.134 ± 1.034	3.019 ± 0.987	3.240 ± 1.252	0.580^†^
Cancelled cycles	0 (0%)	3 (1.92%)	1 (0.64%)	0.626^‡^
Retrieved oocytes	6.13 ± 4.13	6.26 ± 3.36	6.25 ± 3.06	0.880^§^
GQ oocytes	3.50 ± 2.22	3.97 ± 2.44	4.25 ± 2.22	0.476^§^
Quality of embryos				
Top	2.38 ± 1.56	2.90 ± 1.92	2.50 ± 1.54	0.578^§^
Good	1.90 ± 1.60	2.02 ± 1.29	1.47 ± 0.61	0.306^§^
Poor	2.00	1.54 ± 0.88	1.50 ± 0.71	NA
Bad	1.00	1.00	1.00	NA
Transferred embryos	2.13 ± 0.89	1.93 ± 1.15	2.22 ± 0.93	0.589^§^
Total No of embryos	4.17 ± 1.83	3.67 ± 2.51	3.94 ± 2.01	0.706^§^
Without ET	1 (6.25%)	19 (17.11%)	2 (6.89%)	0.502^‡^
ET Cleavage (day2)	9 (56.25%)	58 (52.25%)	16 (55.17%)	0.502^‡^
	6 (37.5%)	27 (24.32%)	7 (24.13%)	
ET Blastocyst stage	0 (0%)	3 (2.7%)	2 (6.89%)	
Biochemical Pregnancy Rate	5 (31.25%)	45 (40.54%)	13 (44.82%)	0.298^‡^
Clinical Pregnancy Rate	5 (31.25%)	43 (38.73%)	11 (37.93%)	0.770^‡^
Ongoing Pregnancy Rate	5 (31.25%)	39 (35.13%)	11 (37.93%)	0.822^‡^

^a^Number with % in brackets or mean ± SD or %. ^†^ANOVA. ^‡^Chi-square. ^§^Kruskal-Wallis test. *NA* non applicable

Despite significant rise in the average age of patients between study groups, being the lowest in underweight and the highest in overweight group (Tables 1 and 3), the rates of biochemical, clinical, and ongoing pregnancy increase in the same manner, although not significantly. Since the study groups significantly differed according to age, logistic regression analysis with primary study outcomes (biochemical, clinical and ongoing pregnancy rates) as the dependent outcomes, and BMI and age as the independent variables, was performed. The analysis revealed that BMI does not significantly affect primary outcomes when adjusted for age. Since stimulation duration was the only secondary study outcome with significant difference between study groups, the same analysis was performed for stimulation duration and it was shown that BMI is still a statistically significant predictor when adjusted for age (*p* = 0.021).

## Discussion

Endometriosis is disease with enigmatic etiology and an anticipated prevalence of 10%, and it can result considerable morbidity, and it is associated with risks for several major chronic diseases, psychological disorders and infertility [9, 10]. Our preliminary study evaluated the effects of BMI on IVF outcomes in non-obese endometriosis patients. To the best of our knowledge, this is the first study to address this issue in a very homogenous group of patients where the all other infertility causes were excluded, and where the diagnosis of endometriosis had been previously established solely during laparoscopy. Preliminary evaluation suggests that infertile women with endometriosis regarding BMI do not differ significantly in IVF outcomes. Nevertheless, a certain differences exist and although some did not reach statistical significance, ones deserve to be thoroughly annotated.

The prevalence of endometriosis as a single infertility factor among IVF couples was 5.61%. Apparently, the stringent selection study criteria led to the decrease of the number of evaluated endometriosis patients, thus explaining why this prevalence is lower than 9%, as described by other authors [11]. The only study that provided data on the prevalence of under, normal and overweight endometriosis patients who underwent IVF procedure [12], together with studies which revealed the prevalence of those BMI groups in general population of infertile women [13] or population undergoing IVF [11, 14] were published over 15 years ago. The prevalence of underweight infertile women irrespective to infertility causes was 3%, normal weight 17% and overweight 42% [13]. However, the same prevalence among infertility patients who underwent IVF in France were 21.8, 55.8, and 10.3% respectively [14], while in Australia were 12.3, 53.26, and 22.69% respectively [11]. Among non-obese endometriosis patients who underwent IVF in Portugal 21.22% were underweight, 59.4% normal weight and 19.54% were overweight women [12]. However, today’s lifestyle and behavior choices are often sedentary and unhealthy and consequently could lead to regrouping of women between populations of underweight, normal weight, overweight and obese women. We delivered up-to-date information on the specific prevalence of those groups among non-obese infertile women with endometriosis undergoing IVF: underweight, normal weight and overweight participants accounted for 10.26, 71.5 and 18.58% of study population, respectively. One of the aims of preliminary evaluation studies is to provide up-to-date data required for the future prospective trials [15]. The importance of such data lies in fact that an estimate of prevalence is needed for sample size calculation, especially under such circumstances where prevalences differ considerably [16]. Since the literature often delivers several different prevalences, up-to-date facts from the most recent preliminary studies with similar study design and population are most preferable [16]. Therefore, future investigators could calculate sample size according to planed primary study endpoint, estimation of pregnancy rates and on those grounds to appraise the time needed to enroll the target number of participants and the overall duration of the trial [15].

Analysis of characteristics of study participants demonstrated several significant dissimilarities between study groups. Underweight and normal weight patients more frequently reside in urban settlements, while overweight patients more often inhabit rural areas. This is in accordance with the study performed in the general population of women, which additionally noted that the rate of overweight increase is greater in rural areas than in urban areas [17]. Moreover, the mean age according to the study groups tends to increase as BMI increases (*p* < 0,001). Missmer et al. observed that the incidence rates of laparoscopically confirmed endometriosis are inversely associated with age [18]. This finding, together with our results which showed that the higher grades of endometriosis are more often present in patients with lower BMI, explains the highly significant differences in age among the study groups, with higher prevalence of moderate and severe forms of the disease among women with lower BMI and vice versa. Furthermore, BMI displayed an inverse gradient from less to more educated groups (*p* = 0,002) which is in line with the study of Lassale et al. [19].

Underweight participants more frequently have grades III and IV of the disease, while overweight patients more frequently have grade I endometriosis (*p* = 0,021). This is in the line with the findings of the majority of other authors [4, 12, 18]. Furthermore, Missmer et al. showed that BMI was associated with the incidence of endometriosis [18], while Moini et al. consider that BMI may be regarded as predictive factors not only for any type of endometriosis but also for severe ones [4]. In contrast, Hemmings et al. did not show any significant correlation between BMI and endometriosis [20]. However, they had different inclusion criteria in terms of a broad spectrum of preoperative indications (infertility, pelvic pain, pelvic mass, and others) and applied type of surgery (laparoscopy/laparotomy, tubal ligation/reanastomosis, hysterectomy). Besides, BMI showed a highly significant inverse correlation with endometriosis grade as infertile women with lower BMI tend to have the more severe form of the disease (*p* < 0.001). This finding was in agreement with studies by Calhaz-Jorge et al. [12], an Italian group [21] and Hediger et al. [22] study and could be explained with the fact that the severity of endometriosis is correlated with peripheral body fat distribution [5]. Underweight and normal weight patients had less frequently extensive surgical treatment (cystectomy of the endometriomas) comparing with overweight patients, due to fall in prevalence of moderate and severe endometriosis grades with increasing BMI.

The doses of used gonadotropins did not significantly differ between study groups. In contrast, most studies agree that the increase in BMI is related with the increased amount of gonadotropins used in the process of COS [1]. While some studies find the significant difference in the quantity of the used gonadotropins in both antagonists, as well as in agonist protocol [23], the other finds this significance only in antagonist protocol [14]. Unlike our study, the mentioned studies evaluated the impact of BMI on the applied amount of gonadotropins among women with different causes of infertility. This may indicate the different impact of BMI on ovarian response to COS in endometriosis patients, particularly if one takes into account that required gonadotropin doses per follicle is significantly higher in endometriosis compared to the women with tubal infertility [24]. The mean stimulation duration differed significantly according to the BMI groups, being highest in the overweight group, which is in line with study of Fedorcsák et al. [25] and Dokras et al. [26], but in contrast to other studies [14, 23]. Still, Wittemer et al. performed study in general population of women undergoing IVF [14], while Marci et al. had endometriosis and type 1 diabetes as exclusion criteria [23].

Several reasons necessitate assessment of BMI influence on IVF outcome among non-obese women with endometriosis. The largest number of women undergoing IVF falls into the category of non-obese women. Besides, endometriosis is inversely related with early adult BMI, unlike most other infertility causes, in which higher BMI decreases reproductive chances [5]. Finally, understanding the impact of BMI on the IVF outcomes in endometriosis women would allow counseling of patients regarding the achievement of ideal BMI prior to the procedure, as part of the individual approach in the infertility treatment.

The previous studies demonstrated negative correlation between BMI and the number of oocytes retrieved in general population of women undergoing IVF [14, 27, 28]. Furthermore, even when divided into underweight, normal weight, overweight group, the underweight women have more oocyte retrieved [28]. Still, our results aroused from the analysis of IVF treatment of women with endometriosis as sole infertility factor were unable to confirm this relationship. Paradoxically, underweight women, who were significantly younger then overweight and normal weight patients, had the lower mean number of retrieved oocytes, although this difference did not reach statistical significance. Possible explanation could be higher prevalence of stage III and IV endometriosis observed among underweight participants compared to other study groups. Women with stage III-IV of endometriosis have fewer oocytes retrieved compared to women with stage I-II of the disease [29].

Although significant differences were not found between the groups in the number of good quality oocytes, total number of embryos, number of GQ embryos and number of transferred embryos, these figures were higher with the increase of BMI. This is not in accordance with Wittemer et al. who observed significantly lower number of good quality oocytes in overweight and underweight patients comparing to normally weighted women [14]. Significantly lower number of our previously surgically treated participants with higher BMI, together with detrimental relationship between endometriosis with previous ovarian surgery and ovarian reserve reported by Matalliotakis et al. [30], could explain our results. Furthermore, inferior ovarian response is more likely present among the subjects with grade III and IV endometriosis [31, 32], which were significantly more prevalent among our participants with lower BMI. Nevertheless, additional explanation of our findings is necessary since Opøien et al. did not find differences in the fraction of mature cumulus-oocyte complexes between ASRM III-IV and ASRM I-II groups [29].

Significant differences in biochemical, clinical and ongoing pregnancy rates between BMI groups were not found, which is in line with the other studies [14, 33, 34], but in contrast to the study of Marci et al. who found higher clinical pregnancies rates in patients with normal BMI comparing to overweight women [23]. When examined as a main variable alone, BMI does not appear to have a significant effect on IVF outcomes, but BMI x age interaction analysis reveals a marked decrease in pregnancy rates with increasing BMI for patients younger than 27 years of age [35]. Surprisingly, although did not reach the statistical significance, our pregnancy rates were higher with increased BMI values, despite the fact that the average age was the lowest in underweight and the highest in overweight participants.

The strength of the current study is the cautiously chosen homogeneous group of patients, which lessens potential confounders. Furthermore, participants showed fertility difficulties before laparoscopic surgery, consequently fertility problems were not necessarily due to the possible effect of surgery. Besides, endometriosis patients undergoing IVF in tertiary referral centers (as our clinic) usually do not differ from endometriosis patients in non-referral centers and general practices and in this sense external validity of the study is fulfilled. Moreover, we initially addressed the topic where no study has ever been done before and delivered information for further definitive studies and provided up-to-date data, needed for the calculation of the sample size for the future main trials. Additionally, contemporary medicine is focused both on the ideal therapeutic approach and on counseling the patient towards behaviors that optimize the effect of applied treatment. The example is the advice to obese and overweight women for the reduction of BMI prior to IVF treatment to achieve the higher pregnancy rates. However, this recommendation stemmed from studies performed in general population of infertile women. We often advise patients according to known facts concerning the general population of women, regardless to characteristics of a patient or a disease. Simultaneously, modern medicine favors customized approach, both in relation to the patient and in accordance to the type or severity of a disease or condition (e.g. endometriosis and infertility). For that reason, it is important to test if facts established for general populations are proper for each type of patient or infertility cause. Future prospective studies could give answer whether it is appropriate to advice overweight women with endometriosis to lose weight prior to IVF, which we often routinely do? Today we are aware that weight related issues, food and nutrients influence pathogenesis and progression of endometriosis. Therefore, dietary practices and lifestyle behaviors are becoming alternative and adjuvant treatments to combat the disease and its consequences, such as infertility [36].

We are aware that the strict inclusion criteria applied lead to a relatively small number of underweight participants and we acknowledge this as limitation of our study. Nevertheless, the study sample is highly homogenous and almost all confound factors that could lead to bias are therefore eliminated. Limitation of the study could be retrospective design, with issues as different assessment of the pelvis and the stage of the disease, different surgical and IVF approach to endometriosis patients. Nevertheless, participants underwent all procedures in our clinic, where the stuff of the Laparoscopy and IVF departments was stable during the study period and where the procedures for endometriosis patients were not considerably changed over the analyzed period. Besides, disease was staged by the same classification system. Further limitation is related to the fact that BMI should not be the single indicator of weight related health and reproductive issues. It does not distinguish android and gynaecoid fat distribution or regional fat distribution. Still, BMI represents a simple and consistent measure that has been individually and steadily related with numerous clinical endpoints.

## Conclusions

Our preliminary results do not support the hypothesis that higher BMI among non-obese endometriosis patients improve IVF outcomes. However, the increase in BMI did not adversely affect the outcomes of IVF either, which is in contrast to literature data as regards general population of infertile women undergoing IVF. Perceived differences between our preliminary data and most of the literature data refer to important IVF outcomes, such as gonadotrophin doses, number of retrieved oocytes, retrieved GC oocytes, total number of obtained embryos, number of GQ embryos, and achieved pregnancy rates. Therefore, we believe that careful consideration of our results could initiate further evaluation of this topic. Prospective studies with large number of patients with endometriosis or prospective case-control studies should address these issues and provide more comprehensive counseling of infertile endometriosis patients regarding achievement of optimal BMI prior to IVF with the intention of achievement higher pregnancy rates.

## Abbreviations

AFC: Antral follicle count
AMH: Anti-Müllerian hormone
ANOVA: Analysis of variance
ASRM: The American Society of Reproductive Medicine
BMI: Body mass index
COS: Controlled ovarian stimulation
E_2_: Estradiol
ET: Embryo transfer
FSH: Follicle-stimulating hormone
GnRH: Gonadotropin-releasing hormone
GnRH-a: GnRH agonist
GnRH-an: GnRH antagonist
GQ: Good quality
hCG: human chorionic gonadotropin
IVF: In vitro fertilization
